# Multipartite entanglement criterion via generalized local uncertainty relations

**DOI:** 10.1038/s41598-021-89067-w

**Published:** 2021-05-05

**Authors:** Jia-Bin Zhang, Tao Li, Qing-Hua Zhang, Shao-Ming Fei, Zhi-Xi Wang

**Affiliations:** 1 School of Mathematical Sciences, Capital Normal University, Beijing, 100048 China; 2 School of Mathematics and Statistics, Beijing Technology and Business University, Beijing, 100048 China

**Keywords:** Quantum information, Quantum physics

## Abstract

We study the detection of multipartite entanglement based on the generalized local uncertainty relations. A sufficient criterion for the entanglement of four-partite quantum systems is presented in terms of the local uncertainty relations. Detailed examples are given to illustrate the advantages of our criterion. The approach is generalized to general multipartite entanglement cases.

## Introduction

Quantum entanglement is a remarkable feature in quantum physics^1^ and has attracted much attention in recent years. Entangled states are recognized as the essential resources in quantum information processing, with many experimental realizations^2,3^ and applications in such as quantum algorithms^4^, quantum teleportation^4,5^, quantum cryptography^6^. Recently, it was shown that quantum entanglement is tightly connected to wave-particle duality, and it can create a wave-particle entangled state of two photons^7^. Detecting entanglement of multipartite systems is a fundamental problem in the theory of quantum entanglement. Separability criteria to determine whether a given state is separable or not are of crucial importance^8^. Enormous efforts have been dedicated to solve the separability problems^9–37^. Nevertheless, the characterization and quantification of multipartite entanglement are less understood than that of bipartite case, as multipartite states can be entangled in more different ways.

There have been many efficient entanglement criteria such as local uncertainty relations (LUR)^11,12^, covariance matrix criterion (CMC)^13^, computable cross-norm or realignment criterion (CCNR)^14^, permutation separability criteria^15^, criterion based on Bloch representations^17,18^, entanglement witnesses^21^, Bell-type inequalities criteria^22^, and criterion based on quantum Fisher information^23^. Generally, these criteria are only necessary condition for separable states and have different advantages in detect different entanglements.

The LUR criterion, the symmetric CMC criterion and the realignment criterion are usually considered as complementary to the the positive partial transposition criterion. The main advantage of LUR criterion is that it allows us to detect the entanglement of quantum states without having to fully understand them, and it can detect bound entangled states more effectively.

Recently, based on the local sum uncertainty relations, some entanglement criteria have been proposed for both discrete and continuous variable bipartite systems and three-qubit systems^31–33^. Zhang et al. proposed a tighter form of the original LUR criterion to improve the range of entanglement detection^31^, Akbari-Kourbolagh and Azhdargalam generalized the LUR criterion to the tripartite systems^33^.

This paper is structured as follows. We start by introducing the entanglement criterion based on LUR for tripartite systems and generalize the entanglement criterion to four-partite quantum systems. Some detail examples are then given to illustrate the advantages of the criterion. Then, the entanglement criterion for *N*-partite systems ($N>4$) is discussed. Brief discussion and summary are given at last.

## Results

Let ${\mathcal {H}}={\mathcal {H}}_1\otimes {\mathcal {H}}_2\otimes \cdots \otimes {\mathcal {H}}_N$ be an *N*-partite system with ${\mathcal {H}}_k$ the $d_k$-dimensional vector space associated with the *k*-th subsystem. An *N*-partite state $\rho \in {\mathcal {H}}$ is said to be separable if $\rho$ can be written as

1 $$\begin{aligned} \rho =\sum \limits_{i} p_i {\rho_i^1}\otimes \rho_i^2\otimes \cdots \otimes {\rho_i^N}, \end{aligned}$$

where ${\rho_i^k}$ are density matrices of the subsystem ${\mathcal {H}}_k$, $0 \le p_i\le 1$, $\sum \limits_i p_i=1$.

In quantum theory, the observables of a quantum system are represented by a set of Hermitian operators $\{A_i\}$. The uncertainty principle shows that it is impossible to predict the measurement results of all observables of the system at the same time. The variance of $A_i$ with respect to $\rho$ is the uncertainty of an observable $A_i$, defining as $(\Delta A_i)^2_\rho =\langle A_i^2\rangle_\rho -\langle A_i\rangle ^2_\rho$, where $\langle A_i\rangle_\rho =\text {Tr}(\rho \, A_i)$ is the mean value. For a set of quantum observables $\{A_i\}$, there exits a constant *U* such that $\sum \limits_{i}{(\Delta A_i)^2_\rho} \ge U$. This inequality gives a universally valid limitation of the measurement outcomes. Generally, it is difficult to determine the value *U*. For the case of Pauli matrices $\sigma_x$, $\sigma_y$ and $\sigma_z$, one has ${(\Delta \sigma_x)^2_\rho} +{(\Delta \sigma_y)^2_\rho} +{(\Delta \sigma_z)^2_\rho} \ge 2$^32^.

In Ref.^33^, based on the local sum uncertainty relations, an entanglement criterion has been presented for tripartite systems.

Let $\{A_1^i\}$, $\{A_2^i\}$ and $\{A_3^i\}$ be the set of local observables associated to the subsystems ${\mathcal {H}}_1$, ${\mathcal {H}}_2$ and ${\mathcal {H}}_3$, respectively. $U_1,~U_2,~U_3$ are lower bound of these local observables, such that $\sum \limits_{i} \Delta {({{A}_2^i})^2} \ge U_{1}$, $\sum \limits_{i}\Delta {({{A}_2^i})^2} \ge U_{2}$ and $\sum \limits_{i}\Delta {({{A}_3^i})^2} \ge U_{3}$. For any separable tripartite states, the following inequalities hold under any permutations of $\{1, 2, 3\}$^33^:

2 $$\begin{aligned} {F_{\rho }^{{1}{2}|{3}}}\equiv \sum \limits_{i}\Delta ({A_{1}^i} + {A_{2}^i}+ {A_{3}^i})_\rho ^2 - (U_{{1}}+U_{{2}}+U_{{3}}+{M_{{1}{2}}^2}+{M_{{1}{2}|{3}}^2})\ge 0, \end{aligned}$$

where ${M_{{1}{2}}}=\sqrt{\sum \limits_i \Delta (A_{1}^i)^2-U_{{1}}}-\sqrt{\sum \limits_i \Delta {(A_{2}^i)^2}-U_{{2}}}$, ${M_{{1}{2}|{3}}}=\sqrt{F^{{1}{2}}_\rho }-\sqrt{\sum \limits_i\Delta {(A_{3}^i)^2}-U_{{3}}}$, ${F_\rho^{{1}{2}}} =\sum \limits_i \Delta ({A_{1}^i}+{ A_{2}^i)^2}-(U_{1}+U_{2}+{M_{{1}{2}}^2})$, ${A_{1}^i}$, ${A_{2}^i}$ and ${A_{3}^i}$ are the operators acting on the first, the second and the third subsystem with the rest subsystems as identity operators in the tripartite systems, respectively.

Generalizing the criterion (2) to four-partite systems, we consider the set of local observables $\{A_1^i\}$, $\{A_2^i\}$, $\{A_3^i\}$ and $\{A_4^i\}$ associated to the subsystems ${\mathcal {H}}_1$, ${\mathcal {H}}_2$, ${\mathcal {H}}_3$ and ${\mathcal {H}}_4$, respectively. From the local sum uncertainty relations, there must exists lower bounds $U_j>0$ for each nonsimultaneous observable $\{A_j^i\}$ for $j=1,2,3,4$. That is to say,

3 $$\begin{aligned} \sum \limits_{i} \Delta {({A}_2^i)^2} \ge U_1, \quad \sum \limits_{i}\Delta (A_2^i)^2 \ge U_2, \quad \sum \limits_{i}\Delta {({A_3^i})^2} \ge U_3,\quad \sum \limits_{i}\Delta {({A_4^i})^2} \ge U_4. \end{aligned}$$

Then for four-partite quantum systems, we have the following conclusion.

### Theorem 1

*For any four-partite separable states, the following inequalities hold simultaneously under any permutations of *$\{1,2,3,4\}$,

4 $$\begin{aligned}{}&F_{\rho }^{{123|4}} = F-(M_{{12}}^{2}+ M_{{12|3}}^{2}+M_{{123|4}}^{2} ) \ge 0, \\&F_{\rho }^{{12|34}}= F-(M_{{12}}^{2}+M_{{34}}^{2}+ M_{{12|34}}^{2} )\ge 0,\\\end{aligned}$$

*where* $$F=\sum \limits_{i}{{\Delta (A_1^i+A_2^i+ A^i_3 + A_4^i)}_\rho ^2}-\sum \limits ^4_{j=1}U_j$$, $$M_{123|4}=\sqrt{F^{12|3}_\rho } -\sqrt{\sum \limits_i\Delta {(A_{4}^i)^2}-U_{4}}$$, $${M}_{12|34}=\sqrt{F^{12}_\rho } -\sqrt{F^{34}_\rho }$$.

*Theorem* 1*provides a necessary condition of separable four-partite states. The violations of the inequalities in* (1) *sufficiently imply entanglement. For the four-qubit* *W* *state,* $\rho =|W_4\rangle \langle W_4|$ *with* $|W_4\rangle =\displaystyle \frac{1}{2}(|1000\rangle +|0100\rangle +|0010\rangle +|0001\rangle )$. *Let* $A_1^1= A_2^1=A_3^1=-A_4^1=\sigma_x$, $A_1^2= A_2^2=A_3^2=-A_4^2=\sigma_y$ *and* $A_1^3 =A_2^3=A_3^3=-A_4^3=\sigma_z$, *thus we get* $\sum \limits_{i}\Delta {({A_j^i})^2} \ge 2$, $M_{12}=0$, $M_{34}=0$, $M_{12|3}=\sqrt{3}-\sqrt{\frac{3}{4}}$, $M_{123|4}= \sqrt{\frac{27}{4}-M^2_{12|3}}-\sqrt{\frac{3}{4}}$ *and* ${M}_{12|34}=\sqrt{3}$, *which give rise to* $F_{\rho }^{123|4}=3-M^2_{12|3}-M^2_{123|4}<0$ *and* $F_{\rho }^{12|34}= 0$, *which provide a violation for the inequalities * (4). *Therefore, the criterion identifies four-qubit* *W* *state is entangled. By taking use of Theorem *1, *more generally states can be detected and we consider some detailed examples for mixed states below.*

### Example 1

**Figure 1:**
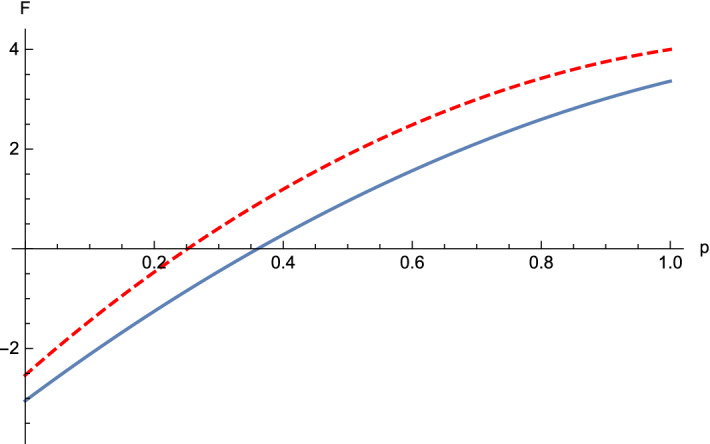
For the four-partite *W* state mixed with the white noise $\rho_1$. The the blue line stands for $F^{123|4}_{\rho_1}$ and the red dash line stands for $F^{12|34}_{\rho_1}$ in Theorem 1. We can see that when $p\le 0.3605$, state $\rho_1$ violates one of the inequalities (4), hence $\rho_1$ is entangled for $p\le 0.3605$.

**Figure 2:**
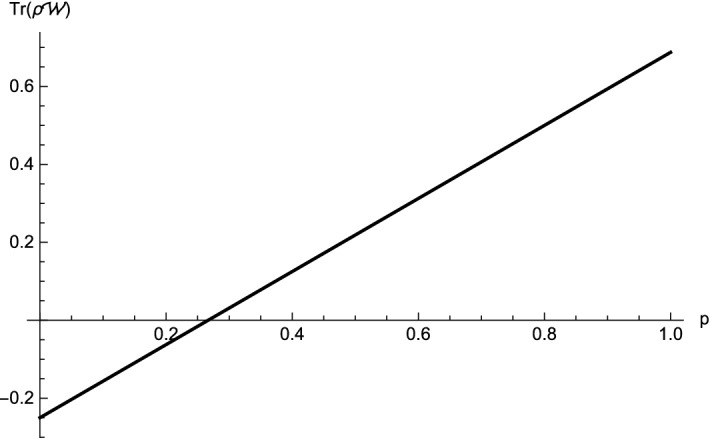
For the four-partite *W* state mixed with the white noise $\rho_1$. The the black line represents $\text {Tr}(\rho_1{\mathcal {W}})$ in Ref.^27^. We can see that $\rho_1$ is detected by the witness $\frac{3}{4}I-|W_4\rangle \langle W_4|$, thus $\rho_1$ is entangled for $p\le 0.267$.

(Four-qubit *W* state mixed with white noise)   We first consider $\rho_1 =\frac{p}{16}I+(1-p)|W_4\rangle \langle W_4|$, $0\le p \le 1$. For this state, we choose $-A^1_1=-A^1_2=-A^1_3=A^1_4=\sigma_x$, $-A^2_1=-A^2_2=A^2_3=A^2_4=\sigma_y$ and $-A^3_1=-A^3_2=-A^3_3=-A^3_4=\sigma_z$, hence $\sum \limits_{i}\Delta (A^i_j)^2 \ge 2$, $M_{12}=M_{34}=0$, $M_{12|3}=\sqrt{3-p^2}-\sqrt{1-\frac{1}{4}(1-p)^2}$, $M_{123|4}=\sqrt{\frac{10p-9p^2+11}{4}-M^2_{12|3}}-\sqrt{1-\frac{1}{4}(1-p)^2}$ and ${M}_{12|34}=\sqrt{3-p^2}-\sqrt{2p-p^2+1}$. Then, we get $F^{123|4}_{\rho_1}=10p-4p^2-2-M^2_{12|3}-M^{2}_{123|4}$ and $F^{12|34}_{\rho_1} = 10p-4p^2-2-{M}^2_{12|34}$. When $p\le 0.3605$, $F^{123|4}_{\rho_1}\le 0$, so the state $\rho_1$ violates one of the inequalities (4). Therefore, the four-partite LUR criterion identifies the $\rho_1$ as an entangled state, see Fig. 1. While, $\rho_1$ is detected based on the witness ${\mathcal {W}}=\frac{3}{4}I-|W_4\rangle \langle W_4|$ which is proposed in Ref.^27^ when $p<0.267$, see Fig. 2. That is to say our result detects better the entanglement than the criterion of Ref.^27^.

### Example 2

**Figure 3:**
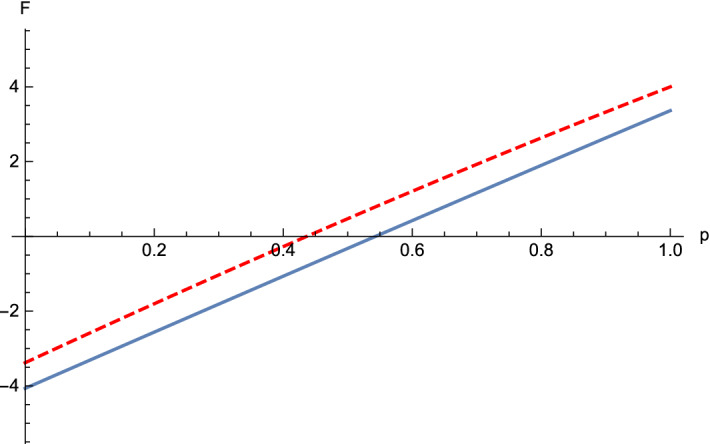
For the four-partite Dicke state $D^4_2$ mixed with the white noise $\rho_2$. The the blue line stands for $F^{123|4}_{\rho_2}$ and the red dash line stands for $F^{123|4}_{\rho_2}$ and the red dash line stands for $F^{12|34}_{\rho_2}$ in Theorem 1. When $p\le 0.3605$, we can see that the state $\rho_2$ violates one of the inequalities (4), whence our criterion detects the entanglement of $\rho_2$ for $0\le p \le 0.543$.

**Figure 4:**
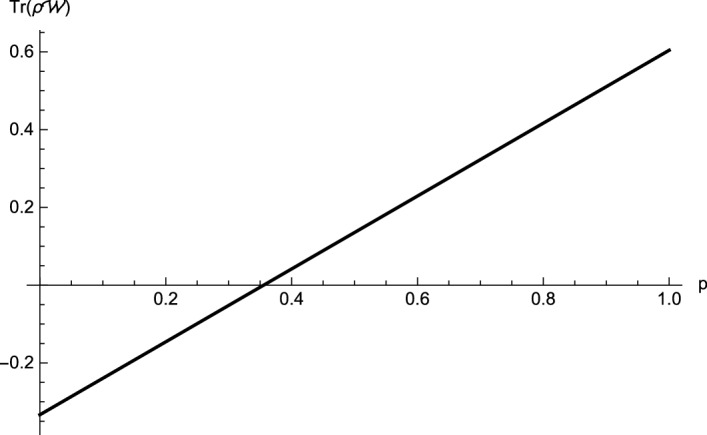
For the four-partite Dicke state $D^4_2$ mixed with the white noise $\rho_2$. The the black line stands for $\text {Tr}(\rho {\mathcal {W}})$ in Ref.^27^. By using the witness ${\mathcal {W}}$, we can see that $\rho_2$ is entangled for $p \le 0.356$.

(Four-qubit Dicke state mixed with white noise)   Now, we take $\rho_2=\frac{p}{16}I + (1-p)(|D^4_2\rangle \langle D^4_2|)$, $0\le p\le 1$, where $|D^4_2\rangle =\frac{1}{\sqrt{6}}(|1100\rangle + |1010\rangle +|1001\rangle +|0110\rangle +|0101\rangle +|0011\rangle )$. For this state, we choose $-A^1_1= -A^1_2=A^1_3=A^1_4=\sigma_x$, $A^2_1= A^2_2=A^2_3=-A^2_4=\sigma_y$, $-A^3_1=-A^3_2=-A^3_3=-A^3_4=\sigma_z$. By direct calculations, we get $M_{12}=0$, $M_{34}=0$, $M_{12|3}= \sqrt{4-2p}-1$, $M_{123|4}= \sqrt{\frac{35}{3}-\frac{26}{3}p-M^2_{123}}-1$ and ${M}_{12|34}= \sqrt{4-2p}- \sqrt{2p}$, which yield $F^{123|4}_{\rho_2}=\frac{22}{3}(p-1)+\frac{2\sqrt{6}}{3}\sqrt{2p-2+3\sqrt{4-2p}}$ and ${F}^{12|34}_{\rho_2}= 8p-8+4\sqrt{\frac{4-p^2}{3}}$. When $p \le 0.437$, $F ^{12|34}_{\rho_2}\le 0$, and $F ^{123|4}_{\rho_2}\le 0$ for $p \le 0.543$. It can be seen, from Fig. 3, that the $\rho_2$ violate inequalities (4) for $p \le 0.543$. Furthermore, comparing with the result in Ref.^27^ which show that $\rho_2$ is entangled for $p < 0.356$ (see Fig. 4), the Theorem 1 also detects more entanglement.

For a more general case, we consider the set of local observable $\{A_1^i\}$, $\{A_2^i\}$, $\ldots$, $\{A^i_N\}$ associated to the subsystems ${\mathcal {H}}_1$, ${\mathcal {H}}_2$, $\ldots$, ${\mathcal {H}}_N$, respectively. Every local observable has a lower bound $U_j$ ($j=1,2,\ldots ,N$) satisfies $\sum \limits_{i} (A^i_j)^2 \ge U_j$. In order to simplify calculation, let $i_N$ represent $\{A^i_{i_N}\}$ and the bi-partition index $(i_1 i_2 \cdots i_K|i_{K+1}\cdots i_N)$ is denoted as ${k_1|k_0}$, where $k_1 =i_1 i_2 \cdots i_K$ and $k_0 =i_{K+1} i_{K+2} \cdots i_N$, $\lceil \frac{N}{2}\rceil \le K<N$ and $1 \le i_1< i_2< \cdots <i_K\le N$. For instance, if $N=4$, hence $K=2$, and $k_1|k_0=\{12|34, 13|24, 14|23\}$, which represents three classes of bi-partition index of local observable set in *N*-body quantum system. Similar to the derivation of the Theorem 1, we obtain the following lemma and theorem.

### Lemma 2

*For multipartite separable states, the following inequalities must hold:*

5 $$\begin{aligned} \sqrt{F^{12\cdots N-1}_\rho }\sqrt{\sum \limits_i \Delta (A_N^i)^2-U_N}\pm \sum \limits_i\Big [\langle (A_1^i+\cdots +A^i_{N-1})\otimes A^i_N\rangle -\langle A_1^i+\cdots +A^i_{N-1}\rangle \langle A^i_N\rangle \Big ] \ge 0, \end{aligned}$$

*and*

6 $$\begin{aligned} \sqrt{F^{k_0}_\rho }\sqrt{F^{k_1}_\rho }\pm \sum \limits_i\Big [\langle (A_1^i+\cdots +A^i_K)\otimes (A^i_{K+1}+\cdots +A^i_N)\rangle -\langle A_1^i+\cdots +A^i_K\rangle \langle A^i_{K+1}+\cdots +A^i_N\rangle \Big ] \ge 0, \end{aligned}$$

*where* $$F^{12\ldots N-1}_\rho =\sum \nolimits_i \Delta (A_1^i+A_2^i+\cdots +A^i_{N-1})^2-(\sum \limits ^{N-1}_{j=1}U_j+M^2_{12}+M^2_{12|3}+\cdots +M^2_{12\cdots N-2|N-1})$$, $${F^{k_0}_\rho } = \sum \nolimits_i \Delta (A_1^i+A_2^i+\cdots +A^i_{K})^2-(\sum \limits ^{K}_{j=1}U_j+M^2_{12}+M^2_{12|3}+\cdots +M^2_{12\cdots K-1|K})$$, $${F^{k_1}_\rho } =\sum \nolimits_i \Delta (A^i_{K+1}+\cdots +A^i_{N})^2-(\sum \limits ^{N}_{j=K+1}U_j+M^2_{K+1 K+2}+\cdots +M^2_{{K+1}{K+2}\cdots N-1|N})$$.

### Theorem 2

*For any multipartite separable states, the following inequalities hold under any permutations of the subsystems,*

$$\begin{aligned} F^{k_1|k_0}_\rho = F-(M^2_{i_1i_2}+M^2_{i_1i_2i_3}+\cdots +M^2_{i_1i_2\cdots i_K}+M^2_{i_{K+1}i_{K+2}}+\cdots +M^2_{i_{K+1}i_{K+2}\cdots i_N}+M^2_{i_1i_2\cdots i_N})\ge 0, \end{aligned}$$

*where*

7 $$\begin{aligned} F=\sum \limits ^N_{i=1} \Delta (A_1^i+A_2^i+\cdots +A^i_N)_\rho ^2- \sum \limits ^N_{j=1}U_j \end{aligned},$$

*and*

8 $$\begin{aligned} \begin{aligned}{}&M_{k_1|k_0}=\sqrt{F^{k_1|k_0}_\rho }-\sqrt{\sum \limits_{i}\Delta (A^i_{i_N})^2-U_{i_N}},~\text {for} ~K=N-1,\\&{M_{k_1|k_0}}=\sqrt{F^{{k}_{1}}_\rho }-\sqrt{F^{k_{0}}_\rho },~\text {for} ~K<N-1. \end{aligned} \end{aligned}$$

$A^i_{i_1}$ *is an operator acting on the* $i_1$-*th subsystem* ${\mathcal {H}}_{i_1}$ *with the rest subsystems as identity operators in* *N*-*partite quantum systems.*

Let us consider five-partite quantum systems to illustrate the theorem. In the case of $N=5$, we can have

$$\begin{aligned} \left\{ \begin{array}{ll} k_1\in \{123,124,125,134,135,145,234,235,245,345\}~~\text {and}~~ k_0\in \{45,35,34,25,24,23,15,14,13,12\} &{} \hbox {K=3;} \\ &{} \\ k_1\in \{1234,1235,1245,1345,2345\} ~~\text {and}~~ k_0\in \{5,4,3,2,1\} &{} \hbox {K=4.} \end{array} \right. \end{aligned}$$

Hence we have

9 $$\begin{aligned} \begin{aligned}{}&F^{1234|5}_\rho = F-(M^2_{12}+M^2_{12|3}+M^2_{123|4}(M^2_{12|34})+M^2_{1234|5}),\\&F^{1235|4}_\rho = F-(M^2_{12}+M^2_{12|3}+M^2_{123|5}(M^2_{12|35})+M^2_{1235|4}),\\&F^{1345|2}_\rho = F-(M^2_{13}+M^2_{13|4}+M^2_{134|5}(M^2_{13|45})+M^2_{1345|2}),\\&F^{2345|1}_\rho = F-(M^2_{23}+M^2_{23|4}+M^2_{234|5}(M^2_{23|45})+M^2_{2345|1}),\\&F^{1245|3}_\rho = F-(M^2_{12}+M^2_{12|3}+M^2_{124|5}(M^2_{12|45})+M^2_{1245|3}),\\&F^{123|45}_\rho = F-(M^2_{12}+M^2_{12|3}+M^2_{45}+{ M}^2_{123|45}),~~F^{124|53}_\rho = F-(M^2_{12}+M^2_{12|4}+M^2_{53}+{ M}^2_{124|53}),\\&F^{125|34}_\rho = F-(M^2_{12}+M^2_{12|5}+M^2_{34}+{ M}^2_{125|34}),~~F^{134|52}_\rho = F-(M^2_{13}+M^2_{13|4}+M^2_{52}+{ M}^2_{134|52}),\\&F^{135|24}_\rho = F-(M^2_{13}+M^2_{13|5}+M^2_{24}+{ M}^2_{135|24}),~~F^{145|23}_\rho = F-(M^2_{14}+M^2_{14|5}+M^2_{23}+{ M}^2_{145|23}),\\&F^{234|51}_\rho = F-(M^2_{23}+M^2_{23|4}+M^2_{51}+{ M}^2_{234|51}),~~F^{235|41}_\rho = F-(M^2_{23}+M^2_{23|5}+M^2_{41}+{ M}^2_{235|41}),\\&F^{245|13}_\rho = F-(M^2_{24}+M^2_{24|5}+M^2_{13}+{ M}^2_{245|13}),~~F^{345|12}_\rho = F-(M^2_{34}+M^2_{34|5}+M^2_{12}+{ M}^2_{345|12}), \end{aligned} \end{aligned}$$

where $F=\sum \limits_{i} \Delta (A_1^i+A_2^i+\cdots +A^i_5)_\rho ^2- \sum \limits ^5_{j=1}U_j$, $M_{1234|5}=\sqrt{F^{123|4}_\rho }-\sqrt{\sum \limits_{i}\Delta (A^i_5)^2-U_{5}}$, ${M}_{123|45}=\sqrt{F^{12|3}_\rho }-\sqrt{F^{45}_\rho }$. $M_{2345|1}$, $M_{1345|2}$, $M_{1245|3}$, $M_{1235|4}$, ${M}_{124|53}$, ${M}_{125|34}$, ${M}_{134|52}$, ${M}_{135|24}$, ${M}_{145|23}$, ${M}_{234|51}$, ${M}_{235|41}$, ${M}_{245|13}$, ${M}_{345|12}$ have similar representations.

As a simple example, consider the five-qubit state $\rho =|W_5\rangle \langle W_5|$, with $|W_5\rangle =\displaystyle \frac{1}{\sqrt{5}}(|10000\rangle +|01000\rangle +|00100\rangle +|00010\rangle +|00001\rangle )$. Let $-A^1_1= A^1_2=-A^1_3=-A^1_4=A^1_5=\sigma_x$, $-A^2_1= -A^2_2=-A^2_3=A^2_4=A^2_5=\sigma_y$, $A^3_1 =-A^3_2=-A^3_3=A^3_4=A^3_5=\sigma_z$. We have $U_1=U_2=U_3=U_4=U_5=2$, $M_{12}=M_{34}=0$, $M_{123}=0.2161$, $M_{123|4}=1.218$, $M_{12|34}=0$, $M_{1234|5}=0.2797$ and $M_{123|45}=0.8536$, which give rise to $F^{1234|5}_{\rho }=3-M^2_{123}-M^2_{1234}-M^2_{1234|5}<0$ and $F^{123|45}_{\rho }<0$, namely, the state is entangled.

## Conclusion

We have generalized the LUR criterion for three qubit quantum systems to multiqubit quantum systems, and obtained new entanglement criteria for four-partite quantum systems as well as for general multipartite systems. By detailed examples we have shown that our criteria can detect better the entanglement than some existing criteria. It is further known that in certain situations they can provide a nonlinear refinement of linear entanglement witnesses^35^, and it can be measured in experimental settings similar to those of entanglement witnesses. The effectiveness of the LUR criteria relies heavily on certain notions of information content of quantum states and choice of observables.

Quantum entanglement is fundamentally connected to the quantum steering, local uncertainty relations (LURs) are a common tool for entanglement detection, and the underlying idea can be directly generalized to steering detection^36^.

The considered system here is closed systems with no decoherence effects taken into account. Also, it would be interesting to find criteria for open quantum systems, since realistic quantum systems inevitably interact with the environment. It would be also interesting if our approach may highlight further investigations on the *k*-separability^37^ of multipartite systems and genuine multipartite entanglement detection.

## Methods

### Proof of the Theorem 1

By straightforward computation, we have

$$\begin{aligned}{}&\sum \limits_{i}\Delta (A_1^i+A_2^i+A^i_3+A_4^i)_\rho ^2=\sum \limits_i \Delta (A_1^i+A_2^i+A^i_3)^2+\sum \limits_i\Delta (A_4^i)^2\\&\quad + 2\sum \limits_i\Big [\langle (A_1^i+A_2^i+A^i_3)\otimes A_4^i\rangle -\langle A_1^i+A_2^i+A^i_3 \rangle \langle A_4^i\rangle \Big ]. \end{aligned}$$

Taking into account that for any tripartite separable states $\rho \in {\mathcal {H}}_1\otimes {\mathcal {H}}_2\otimes {\mathcal {H}}_3$^33^,

10 $$\begin{aligned} \sqrt{F^{12}_\rho }\sqrt{\sum \limits_i \Delta (A_3^i)^2-U_3}\pm \sum \limits_i\Big [\langle ( A_1^i+A_2^i)\otimes A^i_3\rangle -\langle A_1^i+A_2^i\rangle \langle A^i_3\rangle \Big ] \ge 0, \end{aligned}$$

where $F^{12}_\rho =\sum \limits_i \Delta (A_1^i+A_2^i)^2-(U_1+U_2+M^2_{12})$, we obtain

$$\begin{aligned} \sum \limits_{i}\Delta (A_1^i+A_2^i+A^i_3+A_4^i)_\rho ^2 \ge U_1+U_2+U_3+U_4+M^2_{12}+M^2_{12|3}+M^2_{123|4}, \end{aligned}$$

namely, $F^{123|4}_\rho \ge 0$. By relabeling the sub-indices, we have $F^{124|3}_\rho \ge 0$, $F^{134|2}_\rho \ge 0$ and $F^{234|1}_\rho \ge 0$, similarly. Concerning $F^{12|34}_\rho$, we have

$$\begin{aligned}{}&\sum \limits_{i}\Delta (A_1^i+A_2^i+A^i_3+A_4^i)_\rho ^2=\sum \limits_i \Delta (A_1^i+A_2^i)^2 +\sum \limits_i \Delta (A^i_3+A_4^i)^2\\&\quad +2\sum \limits_i\Big [\langle (A_1^i+A_2^i)\otimes (A^i_3+A_4^i)\rangle -\langle A_1^i+A_2^i \rangle \langle A^i_3+A_4^i\rangle \Big ]. \end{aligned}$$

Since for any bipartite separable states $\rho \in {\mathcal {H}}_1\otimes {\mathcal {H}}_2$, the following inequality holds^33^,

11 $$\begin{aligned} \sqrt{\sum \limits_i \Delta {({A}^i_2)^2}-U_1}\sqrt{\sum \limits_i \Delta (A_2^i)^2-U_2} \pm \sum \limits_i\Big [\langle A_1^i\otimes A_2^i\rangle -\langle A_1^i\rangle \langle A_2^i\rangle \Big ] \ge 0, \end{aligned}$$

we get

$$\begin{aligned} \sum \limits_{i}\Delta (A_1^i+A_2^i+A^i_3+A_4^i)_\rho ^2 \ge U_1+U_2+U_3+U_4+M^2_{12}+M^2_{34}+{M}^2_{12|34}, \end{aligned}$$

namely, $F^{12|34}_\rho \ge 0$. Similarly one can show that $F^{23|41}_\rho \ge 0$ and $F^{13|42}_\rho \ge 0$. $\square$

### Proof of the Theorem 2

 We denote the length of $k_0$ as $|k_0|$. From above, one has $|k_0|+|k_1|=N$.

When $K=N-1$, one has $|k_0|=1$, by straightforward computation, we have

$$\begin{aligned}{}&\sum \limits_{i}\Delta (A_1^i+A_2^i+\cdots +A^i_N)_\rho ^2=\sum \limits_i\Delta ({A_1^i}+{A_2^i}+\cdots +A^i_{N-1})^2+\sum \limits_i\Delta (A^i_N)^2\\&\quad +2\sum \limits_i\Big [\langle (A_1^i+A_2^i+\cdots +A^i_{N-1})\otimes A^i_N\rangle -\langle A_1^i+A_2^i+\cdots +A^i_{N-1} \rangle \langle {A_N^i}\rangle \Big ]. \end{aligned}$$

By Lemma 2, for any multipartite separable states $\rho \in {\mathcal {H}}_1\otimes {\mathcal {H}}_2\otimes \cdots \otimes {\mathcal {H}}_N$,

12 $$\begin{aligned}{}&\sqrt{F^{12\cdots N-1}_\rho }\sqrt{\sum \limits_i \Delta (A_N^i)^2-U_N}\nonumber \\&\quad \pm \sum \limits_i\Big [\langle (A_1^i+A_2^i+\cdots +A^i_{N-1})\otimes A^i_N\rangle -\langle A_1^i+A_2^i+\cdots +A^i_{N-1}\rangle \langle A^i_N\rangle \Big ] \ge 0, \end{aligned}$$

via calculation, we obtain

$$\begin{aligned} \sum \limits_{i}\Delta (A_1^i+A_2^i+\cdots +A^i_{N})_\rho ^2 \ge \sum \limits ^N_{j=1}U_j+M^2_{12}+M^2_{12|3}+\cdots +M^2_{12\cdots N-1|N}, \end{aligned}$$

namely, $F^{12\cdots N-1|N}_\rho \ge 0$. By relabeling the sub-indices, we have $F^{k_1|k_0}_\rho \ge 0$.

When $K<N-1$, one has $|k_0|\ge 2$,

$$\begin{aligned}\sum \limits_{i}\Delta (A_1^i+\cdots +A^i_N)_\rho ^2&=\sum \limits_i \Delta (A_1^i+\cdots +A^i_K)^2+\sum \limits_i \Delta (A^i_{K+1}+\cdots +A^i_N)^2\\&\quad +2\sum \limits_i\Big [\langle (A_1^i+\cdots +A^i_K)\otimes (A^i_{K+1}+\cdots +A^i_N)\rangle -\langle A_1^i+\cdots +A^i_K \rangle \langle A^i_{K+1}+\cdots +A^i_N\rangle \Big ]. \end{aligned}$$

By using Lemma 2, we get

$$\begin{aligned} \sum \limits_{i}\Delta (A_1^i+A_2^i+\cdots +A^i_N)_\rho ^2 \ge \sum \limits ^N_{j=1}U_j+(M^2_{12}+M^2_{12|3} +\cdots +M^2_{12\cdots |K}+M^2_{{K+1}{K+2}}+\cdots +M^2_{{K+1}\cdots |N}+M^2_{12\cdots |N}), \end{aligned}$$

namely, $F^{12\cdots K|K+1 K+2\cdots N}_\rho \ge 0$. By relabeling the sub-indices, one can show that $F^{k_0|k_1}_\rho \ge 0$.      $\square$
